# Attempted Bribery of M. Magendie

**Published:** 1847-07

**Authors:** 


*Attempted Bribery of M. Magendie.*—M. Magendie had been appointed as a special witness to give evidence on the question whether certain leeches which had been sold by MM. Vaucher and Laurens were "gorged" * at the time of sale. Madame Laurens, the wife of one of the accused, was charged with attempting to bribe M. Magendie. This gentleman deposed that the accused called on him on the 24th January. After having begged him to devote the greatest care to the delicate investigation entrusted to him, the lady withdrew, leaving upon his table a sealed packet, which she said contained memoranda for his guidance in the case! After her departure, M. Magendie opened the packet, and found that it contained three bank notes of 1000 francs. There was also enclosed a letter, not signed, from which the following extract was read:—'Let me beg of you to procure the dismissal of the complaint against MM. Laurens and Vaucher. This will be simple justice. Your time is valuable, and I therefore wish to remunerate you. No one shall know of my visit to you.'

Madame Laurens said, in her defence, that she had made a mistake in writing the letter; she had not intended to leave it behind her; and if it contained bank notes of 1000 francs, they must have got into the envelope without her knowledge. Her leaving the packet on the table must have been accidental. The tribunal, however, rejected this defence, condemned Madame Lurens to one month's imprisonment, and a fine of 300 francs; and they ordered the notes for 3000 francs, intended as the bribe, to be paid into the account for the benefit of the Parisian infirmaries."—*L'Union Medicale.*

Well done, Madame! but you must proceed more cautiously and prudently in future. You must learn better how to turn away the wrath or to soften the obduracy of those who report on your leeches. In Timbuctoo those who deal in gorged leeches manage things better.—*Dublin Med. Press.*

* In France leeches are sold by weight. It has been much the practice with leech-venders lately to let the leeches fill themselves with blood from calves, horses, and other animals, and sell them in this "gorged" state.

